# Supplementary figures and images for: SiGMoiD: A super-statistical generative model for binary data
Source: PLoS Comput Biol. 2021 Aug 6;17(8):e1009275. doi: 10.1371/journal.pcbi.1009275 (PMC8372922; doi:10.1371/journal.pcbi.1009275)

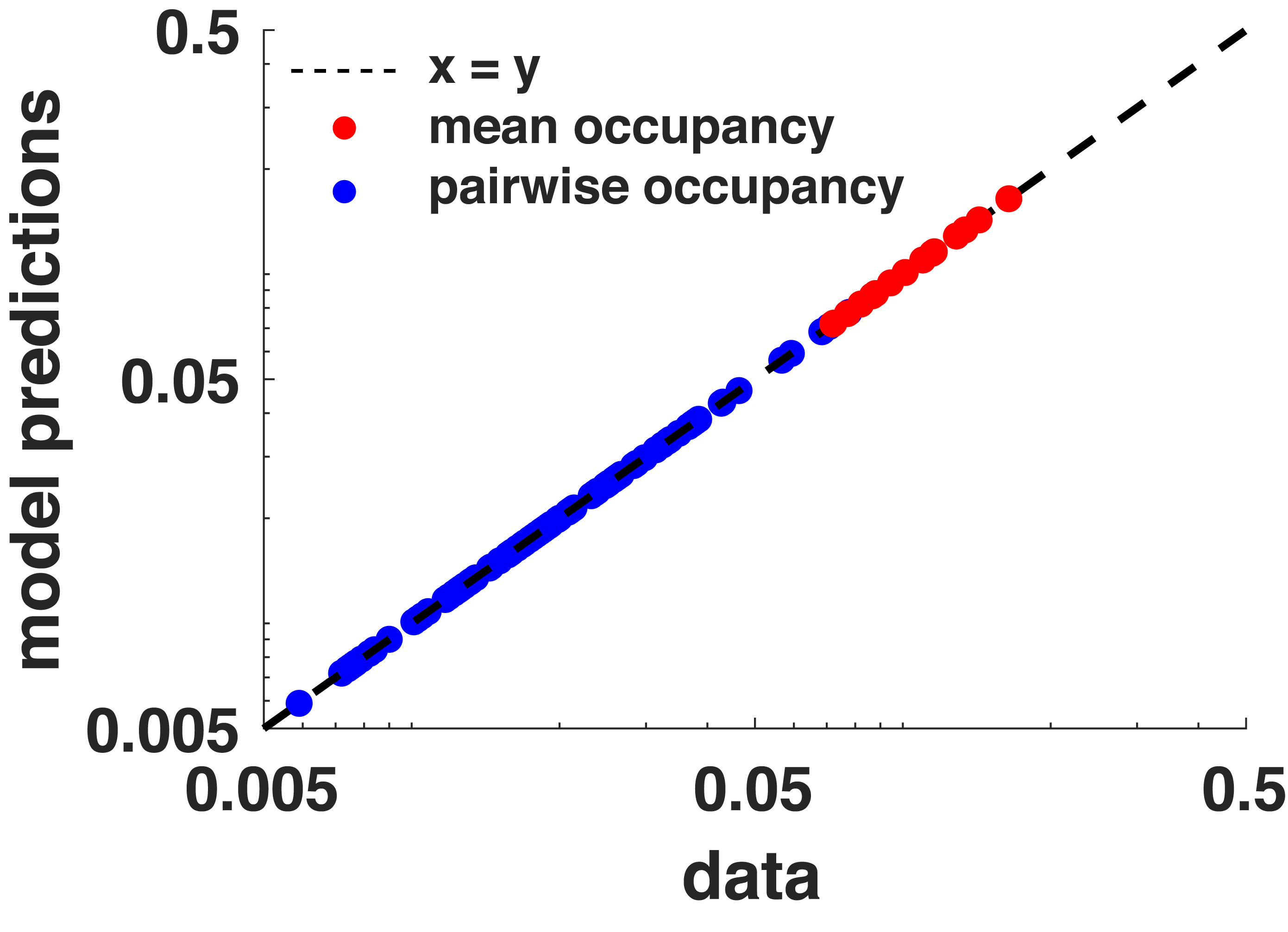

Supplement: S1 Fig — (TIF) [file pcbi.1009275.s001.tif]

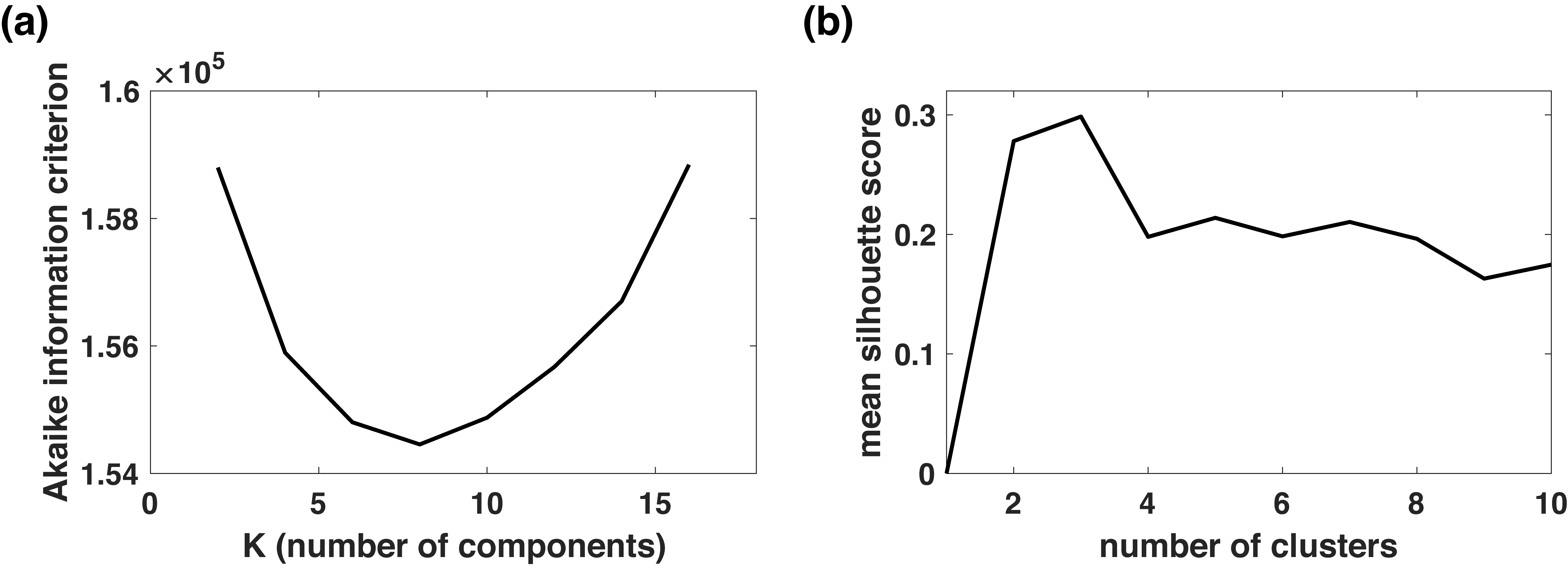

Supplement: S2 Fig — (A) Akaike information criterion as a function of K, the number of components used to model the microbiome co-occurrence data. (B) Mean silhouette score as a number of clusters using K-means clustering of the particle-specific βs latents. (TIF) [file pcbi.1009275.s002.tif]

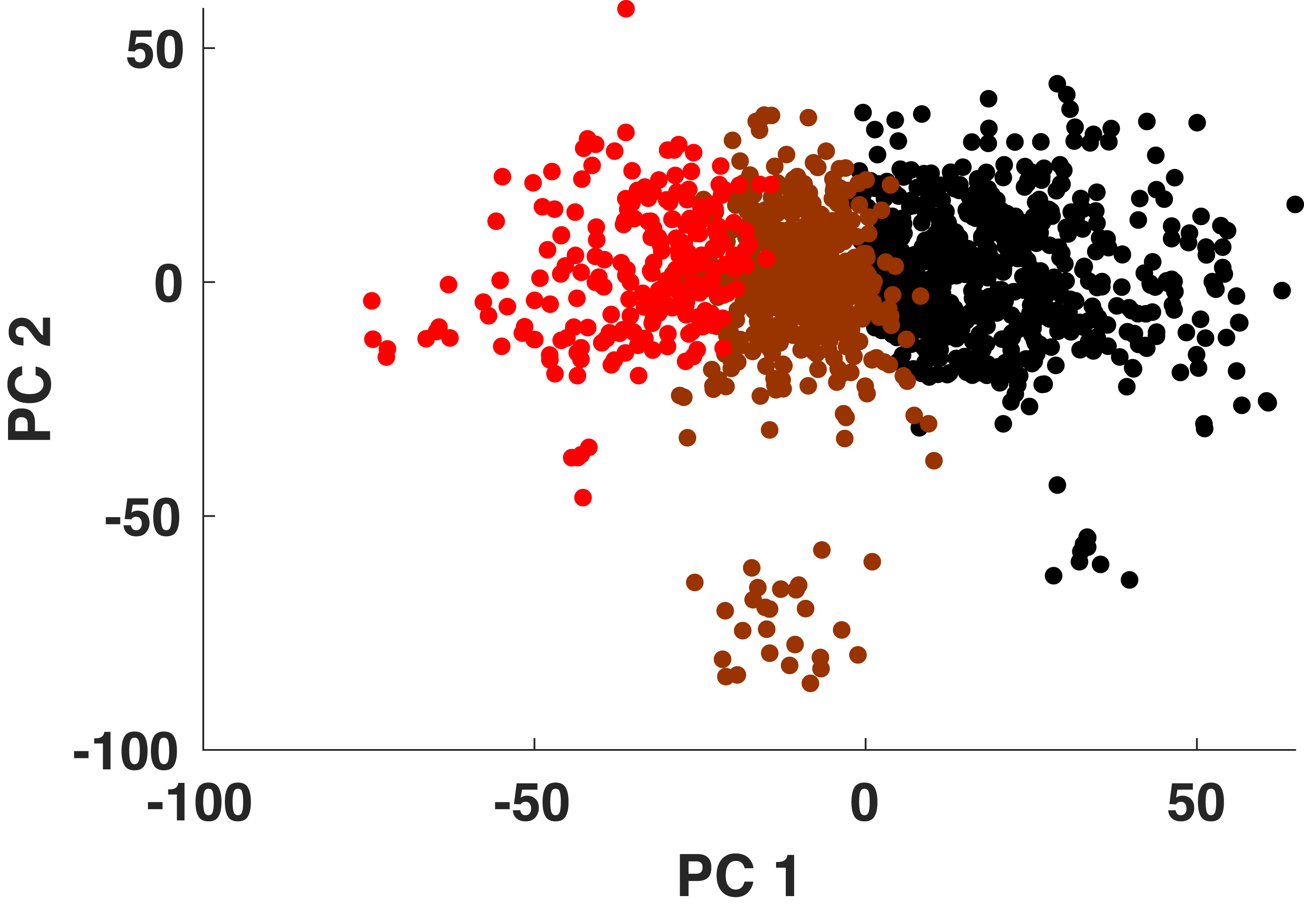

Supplement: S3 Fig — (TIF) [file pcbi.1009275.s003.tif]

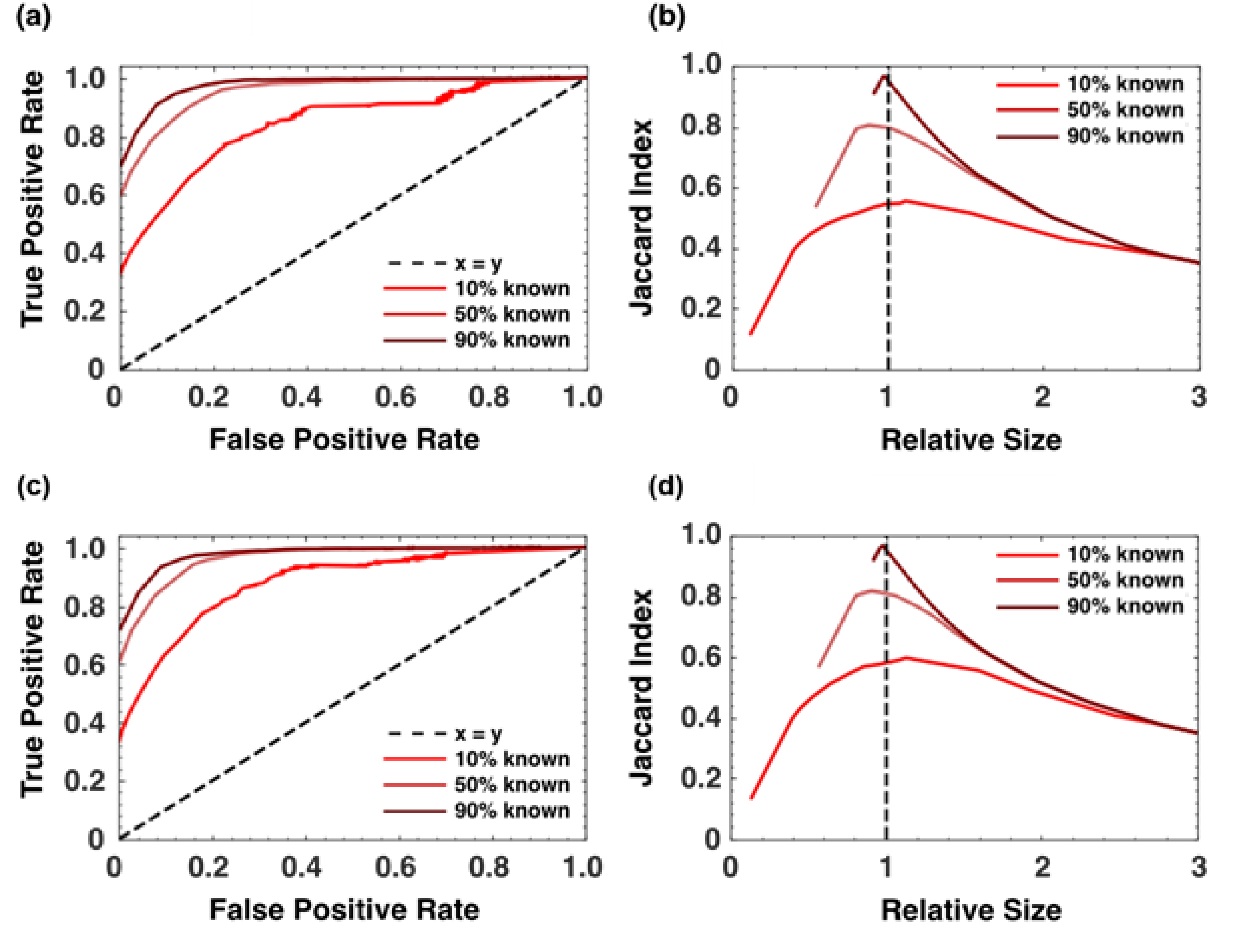

Supplement: S4 Fig — (TIF) [file pcbi.1009275.s004.tif]
