# Supplementary material for: SiGMoiD: A super-statistical generative model for binary data
Source: PLoS Comput Biol. 2021 Aug 6;17(8):e1009275. doi: 10.1371/journal.pcbi.1009275 (PMC8372922; doi:10.1371/journal.pcbi.1009275)
Supplement: S1 Table — (DOCX) [file pcbi.1009275.s005.docx]

**Supplementary Table**

| **Genus** | **Log_10_ p** |
| --- | --- |
| Christensenella | -18.9674 |
| Hyphomonadaceae | -18.0262 |
| Lachnospiraceae | -13.8931 |
| Lachnospiraceae | -13.7517 |
| Lachnospiraceae | -13.3980 |
| Lachnospiraceae | -11.4581 |
| Clostridiales Incertae SedisXII12 | -11.3990 |
| Ruminococcaceae | -10.9595 |
| Ruminococcaceae | -10.4919 |
| Lachnospiraceae | -9.9509 |

**S1 Table.** Genera of OTUs that are most enriched in cluster 3 using a hypergeometric test and the corresponding p-values.
