# Supplementary material for: SiGMoiD: A super-statistical generative model for binary data
Source: PLoS Comput Biol. 2021 Aug 6;17(8):e1009275. doi: 10.1371/journal.pcbi.1009275 (PMC8372922; doi:10.1371/journal.pcbi.1009275)
Supplement: S1 File — (DOCX) [file pcbi.1009275.s006.docx]

**Supplementary Information File**

**Curating the data**

All the data used in this study and the code used to analyze the data can be found on github: https://github.com/zhaoxc099/sigmoid

**Collective firing of neurons**

Neuron firing data was obtained from an open source repository^17,18^. We combined the data across all trials and all time points and selected 15 neurons that had the highest overall firing propensity. We randomly selected ${10}^{4}$ samples from the data for further analysis.

**Co-occurrence of bacterial species**

Bacterial co-occurrence data was downloaded as an OTU table from Sheth et al.^2^. The OTU table was binarized by assigning a 1 if a particular OTU was present (positive abundance) in any given sample and zero otherwise. We removed the OTUs that were present in none of the samples from our analysis.

**Bacterial metabolic networks**

From a list of $\sim27000$ annotated bacterial genomes in the Kbase database^28^ (March 2017) a representative genome was selected for each named species. The representative genome was chosen so that it was close to the median number of genes across all genomes for the corresponding species. Genomes that were obtained using single-cell sequencing were not considered due to the possibility of low genome coverage.

For each selected genome, we built a draft genome-scale metabolic reconstruction using the ‘Build Metabolic Model’ method (v.1.5.1) in the Kbase Narrative interface. The method implements the ModelSeed pipeline described by Henry et al.^29^. We only considered reconstructions with less than 30% reactions added during the gap-filling step of the ModelSeed pipeline as a proxy for high quality models.

The gap-filling approach taken by Kbase involves minimization of an arbitrarily trained optimization function. Therefore, in our analysis we only included reactions that were annotated to a gene or to a combination of genes. At the end, we had a total of $\sim4000$ bacterial metabolic models with a metabolic universe of $\sim3300$ reactions. 400 models were randomly chosen to be in the testing data set and the rest were used to infer SiGMoiD model parameters.
