# Supplementary material for: SiGMoiD: A super-statistical generative model for binary data
Source: PLoS Comput Biol. 2021 Aug 6;17(8):e1009275. doi: 10.1371/journal.pcbi.1009275 (PMC8372922; doi:10.1371/journal.pcbi.1009275)
Supplement: Attachment — Submitted filename: SIGMOID.pdf [file pcbi.1009275.s007.pdf]

Sunday, May 9, 2021

SiGMoiD

“A super-statistical generative model for binary data”

The manuscript “A super-statistical generative model for binary data” by Zhao et al. follows a maximum entropy (max ent) based approach to simulate high dimensional co-varying binary data and infer model parameters based on maximum likelihood methods. Notably, the work addresses some of the shortcomings of max ent methods and important challenge in genomics where the number of covarying quantities are very high; the approach, results and interpretation are scientifically sound; and the manuscript is well written. Nonetheless, I have several major concerns about this work and manuscript that I describe further details below and that do not allow me to recommend for publication at least not in its current form at PLOS Computational Biology.

Specific concerns to be addressed:

More important:

1. The work sounds very general in the sense of the analysis of binary data. However, the restrictions on what data sets are appropriate for the analysis is not explicitly mentioned. Specifically, the analyzed three data sets were subject to post processing prior to the analysis. Therefore, it is not clear how the simulation of data can be carried out for different biological setups to be able to validate the method for different model parameters.
2. It is not clear, besides the embedded model reduction parameter K, what is it making this max ent method going beyond the existing methods. This new method presented in the papers seems more like a reformulation of the existing methods to make clustering easier within a sample (E) and amongst the samples (Beta). More importantly, both methods use gradient descent/ascent algorithms to infer the parameters therefore, apart from the reduced dimension with K, it is not clear what makes the presented method fundamentally different from the existing methods.
3. Even though model reduction seems to be one of the key characteristics of the presented method, the way that the model is reduced does not come with a model uncertainty. Therefore, we would never know the confidence of this model reduction as it is purely relying the maximum likelihood estimates of the model parameters.

Less important:

1. The authors use observation data and samples in an alternating fashion and that makes reading somewhat rough. In a statistics based paper samples first reminds me of the samples that are drawn from the distribution, I suggest they stick to one of them and tell the readers what they mean at the first instance. Specifically, samples appear in the Introduction without specifying it is each observation data set and then in the Figure 1 caption the observation data is explained as samples.

2. In “The model” section the model is presented for one sample although in the cartoon figure the model parameters do not match with the cartoon figure. I suggest the authors

provide their model presentation in the most general form that matches with the rest of the manuscript.

3. The sentence “In contrast, the energies vary from variable to variable but are shared across all samples” sounds confusing. The formulation is built such that the energies (E latent variables to be inferred) are shared across all the analyzed data sets. Therefore, it is not clear what variable that the authors are referring to.
4. After Eq. 6, MCMC based approaches are mentioned although it is not clear what parameters are attempted to be estimated by MCMC based approaches.
5. In Fig. 2, probability of data is provided however the authors do not provide how that is obtained. Moreover, in every panels of Fig. 2, it is not stated in the caption what governs the number of points in every scatter plot.
6. In the section “Inference of interactions from bacterial ..”, the authors do not provide how many particles are analyzed. Similarly, it is not stated how many OTU they look into.
7. In Fig. 3, panel (a), it is not clear how they obtained the red curve as the mean occupancy. Similarly, in panel (b), the rows seem to denote the reduced dimension “K” although there is no label, however, it is very difficult to interpret how many OTU’s are studied.
8. In the end of section “Inference of interactions from bacterial ..” , the authors mention that they could not detect the tightly correlated species when they approach their analysis to the full data. Therefore, it is not clear how many particles they studied and how they selected the analyzed particles.
9. In the last section, they mention that “… given K, we identified groups of reactions with similar occurrence profiles …by clustering E_{ki} values” how is this similarity defined?
10. SigmoID seems to be taken already. Introducing the same acronym does not sound like the most optimized way of naming the computational tool.
