# Supplementary material for: SiGMoiD: A super-statistical generative model for binary data
Source: PLoS Comput Biol. 2021 Aug 6;17(8):e1009275. doi: 10.1371/journal.pcbi.1009275 (PMC8372922; doi:10.1371/journal.pcbi.1009275)
Supplement: Attachment — Submitted filename: SIGMOID_R2.pdf [file pcbi.1009275.s009.pdf]

Sunday, June 27, 2021

SiGMoiD

“A super-statistical generative model for binary data”

The authors modified their originally submitted paper and carefully addressed my questions and comments. Overall, I am satisfied with the answers that they gave and I don’t have further major questions.

Remaining minor concerns:

1 - In lines 259-261, they clearly mention the use of AIC when the data points are not expected to be correlated in time.

Can authors provide a paragraph in how the users should decide for the optimal choice of K when the data points are expected to be correlated in time?

2 - In line 151, did the authors mean to use “batch” instead of “bath” ?
