# Supplementary material for: SiGMoiD: A super-statistical generative model for binary data
Source: PLoS Comput Biol. 2021 Aug 6;17(8):e1009275. doi: 10.1371/journal.pcbi.1009275 (PMC8372922; doi:10.1371/journal.pcbi.1009275)
Supplement: Attachment — Submitted filename: PLOS_reviews.docx [file pcbi.1009275.s008.docx]

Dear Editors,

We thank both the reviewers for their insightful comments. We have now addressed their issues. Below, we provide a point-by-point response.

Thank you

Xiaochuan Zhao, German Plata, and Purushottam Dixit

**Reviewer 1**

The manuscript “A super-statistical generative model for binary data” by Zhao et al. follows a maximum entropy (max ent) based approach to simulate high dimensional co-varying binary data and infer model parameters based on maximum likelihood methods. Notably, the work addresses some of the shortcomings of max ent methods and important challenge in genomics where the number of covarying quantities are very high; the approach, results and interpretation are scientifically sound; and the manuscript is well written. Nonetheless, I have several major concerns about this work and manuscript that I describe further details below and that do not allow me to recommend for publication at least not in its current form at PLOS Computational Biology.

Specific concerns to be addressed:

**More important:**

1. The work sounds very general in the sense of the analysis of binary data. However, the restrictions on what data sets are appropriate for the analysis is not explicitly mentioned. Specifically, the analyzed three data sets were subject to post processing prior to the analysis. Therefore, it is not clear how the simulation of data can be carried out for different biological setups to be able to validate the method for different model parameters.

We thank the reviewer for the important comment. While SiGMoiD will work with any type of binary data, indeed, the data analyzed in this manuscript was binarized in a pre-processing step prior to the analysis. For example, the neural firing data comprises measurements of electrical activity (spiking) that resembles digital signals (firing/silent). Similarly, the microbial presence data was reported as relative abundances which were then binarized. There are several cases where binarization of data is well established: presence/absence of genes in genomes, reactions in metabolic networks, presence/absence of interactions in protein-protein interaction networks, firing/silence of neurons, and so on. We have now clarified this in the manuscript (lines 84-93)

2. It is not clear, besides the embedded model reduction parameter K, what is it making this max ent method going beyond the existing methods. This new method presented in the papers seems more like a reformulation of the existing methods to make clustering easier within a sample (E) and amongst the samples (Beta). More importantly, both methods use gradient descent/ascent algorithms to infer the parameters therefore, apart from the reduced dimension with K, it is not clear what makes the presented method fundamentally different from the existing methods.

We thank the reviewer for raising this important point. We have now clarified in the manuscript the main issues with traditional max ent and the salient features of our approach (lines 95-141).

**Issues with traditional max ent**: **(1)** Perhaps the biggest limitation of traditional max ent is that the modeler is required to *a priori* identify constraints that are appropriate for a given system. Depending on the complexity of interactions, these constraints may not be obvious. A work around is to impose a very large number of constraints. For example, a max ent model to correlated firing of $N$ neurons will typically involve $N$ constraints on mean firing rates and $\sim N^{2}$ constraints on mean co-occurrence frequencies. **(2)** Most often, these max ent models are not analytically solvable and as a result, inference of Lagrange multipliers in these models is computationally infeasible even when the number of dimensions is only moderately large ($N\sim100$). **(3)** The numerical values of the constraints are often evaluated using data which implicitly assumes that data points are statistically independent of each other. This assumption is not true in most practical applications, for example, in temporally correlated firing of neurons or phylogenetically related protein sequences. These limitations together have severely limited the application of max ent when there an increasing interest in describing the collective behavior of thousands of cells, genes or microbial species, among others.

**How we address those issues in SiGMoiD**: **(1)**, in SiGMoiD, the modeler only specifies the total number of constraints. The constraints are optimally learned from the data and a max ent model is fit to those constraints. **(2)** As a result of this optimal data-driven choice of constraints, SiGMoiD requires a much smaller number of constraints than traditional max ent. Consequently, the inference of max ent models is significantly faster than typical max ent, allowing us to analyze very high dimensional data (dimensions $\gg1000$) that remain well out of the reach of current max ent methods. **(3)** SiGMoiD does not assume that sample points are drawn from the same distribution. Instead, motivated by superstatistics, it imagines each data point as arising from its own max ent probability distribution. Since each sample point is approximated by a small set of Lagrange multipliers, SiGMoiD is a non-linear dimensionality reduction method.

3. Even though model reduction seems to be one of the key characteristics of the presented method, the way that the model is reduced does not come with a model uncertainty. Therefore, we would never know the confidence of this model reduction as it is purely relying the maximum likelihood estimates of the model parameters.

The reviewer raises an excellent point. Given the probabilistic nature of our inference, model uncertainties are automatically available in the form of the Bayesian posterior distribution. We have now included a discussion on extending the parameter inference beyond the maximum likelihood estimates to a full posterior distribution over parameters (lines 192-195).

**Less important:**

1. The authors use observation data and samples in an alternating fashion and that makes reading somewhat rough. In a statistics based paper samples first reminds me of the samples that are drawn from the distribution, I suggest they stick to one of them and tell the readers what they mean at the first instance. Specifically, samples appear in the Introduction without specifying it is each observation data set and then in the Figure 1 caption the observation data is explained as samples.

We thank the reviewer for pointing out issues with our language. We have now clearly defined our terminology (variables, sample, data, etc.) and fixed it throughout the manuscript.

2. In “The model” section the model is presented for one sample although in the cartoon figure the model parameters do not match with the cartoon figure. I suggest the authors provide their model presentation in the most general form that matches with the rest of the manuscript.

We thank the reviewer for the suggestion. We have now presented the model in a manner that is consistent between the schematic figure and the text (lines 143-167).

3. The sentence “In contrast, the energies vary from variable to variable but are shared across all samples” sounds confusing. The formulation is built such that the energies (E latent variables to be inferred) are shared across all the analyzed data sets. Therefore, it is not clear what variable that the authors are referring to.

We thank the reviewer for pointing out the issue. We have now clearly defined the various concepts in our approach (variables, samples, data, etc.) (lines 169-171).

4. After Eq. 6, MCMC based approaches are mentioned although it is not clear what parameters are attempted to be estimated by MCMC based approaches.

The MCMC simulations are performed to estimate the gradients used in the determination of the Lagrange multipliers. We have now clarified this issue (lines 222-226).

5. In Fig. 2, probability of data is provided however the authors do not provide how that is obtained. Moreover, in every panels of Fig. 2, it is not stated in the caption what governs the number of points in every scatter plot.

We have now clarified our description (lines 226-233).

6. In the section “Inference of interactions from bacterial ..”, the authors do not provide how many particles are analyzed. Similarly, it is not stated how many OTU they look into.

We have now included these details (lines 263-264).

7. In Fig. 3, panel (a), it is not clear how they obtained the red curve as the mean occupancy. Similarly, in panel (b), the rows seem to denote the reduced dimension “K” although there is no label, however, it is very difficult to interpret how many OTU’s are studied.

The red curve in panel (a) is the null expectation given by the probabilities of occurrence of n OTUs in any given particle calculated using the occurrence frequencies of individual OTUs but neglecting the correlations between OTUs. We have now changed our description and the figures to reflect these changes (lines 285-290).

8. In the end of section !Inference of interactions from bacterial ..” , the authors mention that they could not detect the tightly correlated species when they approach their analysis to the full data. Therefore, it is not clear how many particles they studied and how they selected the analyzed particles.

We thank the reviewer for the comment. Our clustering of the sample-specific beta vectors identified 47 “special” particles (samples). The co-occurrence of several OTUs was much higher in these particles compared to the rest of the particles. Notably, no such cluster was detected when we clustered the samples directly. We have now included these details in the manuscript.

9. In the last section, they mention that “… given K, we identified groups of reactions with similar occurrence profiles …by clustering E_{ki} values” how is this similarity defined?

We have now included these details. Specifically, The similarity $d_{ij}$ between two OTUs $i$ and $j$ was defined as the L2 norm:$d_{ij}^{2}=\sum_{k} \left( E_{ki}-E_{kj} \right)^{2}$ (lines 383-384).

10. SigmoID seems to be taken already. Introducing the same acronym does not sound like the most optimized way of naming the computational tool.

We thank the reviewer for pointing out the previous usage of SiGMoiD. It would have been ideal to choose a different name but now that we have already committed to it, we feel that it is alright to stick with our choice.

**Response to reviewer 2**

Zhao, Plata and Dixit present a novel method to characterize binary data. They apply their method to three experimental datasets and demonstrate its ability to capture nontrivial trends in the context of neuroscience and bacterial populations. Overall, the results seem promising and distinct from the go-to, top-down approach of maximum entropy to model experimental data. I have a few questions about the details of SiGMoiD and how it distinguishes itself from maximum entropy to hopefully clarify SiGMoiD’s novelty in modeling binary data.

1. The authors mention several times that SiGMoiD parameter estimation is much faster than that of maximum entropy. Implicit in this discussion seems to be that maxent methods require Markov chain Monte Carlo (MCMC) simulations to estimate parameters while SiGMoiD can employ gradient ascent. First, why is gradient ascent necessarily faster than MCMC in this case? Second, why can maxent not be cast in the same framework to have parameters estimated by gradient ascent?

We thank the reviewer for raising the point and we apologize for the confusion. The key difference between traditional max ent inference and SiGMoiD is not gradient-based approach but in fact that in SiGMoiD, the gradients can be evaluated analytically. In traditional max ent models for binary data i.e. models constrained to reproduce mean occupancy and covariance constraints, the gradients of the log likelihood of the data with respect to the Lagrange multipliers have to be evaluated using MCMC simulations. In contrast, the SiGMoiD gradients can be evaluated using simple matrix multiplication. We have now clarified this point in the manuscript (lines 95-141).

Could the authors elaborate why maxent using MCMC cannot infer models greater than 100 variables? Are the authors assuming that the maxent model corresponds to one that enforces constraints on the covariances as well as the means?

We thank the reviewer for the comment. Indeed, the reviewer is correct. Traditional max ent models that capture coupled behavior of binary variables typically use mean occupancies and covariances as constraints. The Lagrange multipliers involved in these models cannot be determined analytically and have to be estimated using numerical gradient based methods. The gradients themselves are typically determined using MCMC simulations. Consequently, the inference of these models on > 100 variables is computationally prohibitive. We have now clarified this issue (lines 95-141).

Given that both energies and betas are fitted in SiGMoiD, I would think that there would be a lot of degeneracies in the likelihood, potentially making parameter estimation challenging. Could the authors comment on this potential issue?

The reviewer raises an excellent point. There is significant non-identifiability in our inference. For example, the temperatures can be multiplied by one number and the energies can be divided by the same number without changing the model predictions. We have now explicitly stated these degeneracies in our inference and suggested ways to remove them if required (lines 197-204).

2. Could the authors comment why they limit SiGMoiD to only mean constraints? Why not also look at pairwise constraints, for example? The fact that SiGMoiD currently only encodes mean constraints needs to be delineated in the main text because statements regarding SiGMoiDs ability to infer any constraints from the data are more limited that what a quick read might imply.

We thank the reviewer for the comment and apologize for the confusion. In traditional max ent models of binary data, for example, when modeling collective firing of neurons, one typically imposes constraints on the mean firings $\langle\sigma_{i}\rangle$ of individual neurons as well as $\left\langle\sigma_{i}\sigma_{j} \right\rangle$, the mean values of correlated firings of all pairs of neurons. These constraints are chosen a priori by the modeler.

In contrast, SiGMoiD is not limited to mean constraints i.e. constraints on means of individual neurons. SiGMoiD hypothesizes that the state of each neuron is described by a set of K energy-like variables. We build Max Ent models for individual neurons infer these extensive variables by matching the mean values of these variables. The correlations between the neurons are automatically captured due to the fact that the neuron states in any sample (data point) are described by the same intensive variables.

3. I am curious why SiGMoiD is limited to binary data. What steps in the SiGMoiD pipeline are not possible to numerically compute if samples are not binary?

The reviewer raises an excellent point. In fact, we have another manuscript in review in a different journal where we apply a similar method to model real valued abundance data. We are also developing a generalization of SiGMoiD that is applicable to categorical data; allowing D variables instead of 2 (for example, 4 nucleotides or 20 amino acids). We now describe these possible generalizations in the discussion section (lines 440-452).

More comments:

Have the authors considered comparing SiGMoiD to a standard clustering algorithm? Just as the authors elegantly compared SiGMoiD with maxent based on pairwise correlations, it would be interesting to see how it performs with standard clustering algorithms, given that clustering is a powerful insight provided by SiGMoiD.

We thank the reviewer for the comment. For one of the data sets that we examined (bacterial co-occurrences), one standard clustering approach (K-means clustering) does not identify the small cluster of particles that SiGMoiD identified. We note that clustering the samples is only one of the utilities of SiGMoiD. In addition, SiGMoiD also allows us to identify similarities in the feature space, generate new data, and assign probabilities to unseen data.

Perhaps to make it clear how the maxent model works and better elucidate its difference between SiGMoiD, the authors should demonstrate that pairwise correlations are perfectly captured by the maxent model in Figure 2.

We thank the reviewer for the suggestions. We have now included the pairwise correlations as supplemental figure.

Discussions in the Introduction on modeling neuron firing should include additional citations to William Bialek’s seminal works, such as (Schneidman et al. 2006; Tkačik et al. 2015)

We have now included citation to these papers.

Perhaps the authors would be interested in the following paper, which proposes a method to infer maxent parameters in large systems (Weistuch et al. 2020).

We thank the reviewer for bringing to our attention this manuscript. We have now included a citation to it.

In the Results, the following sentence which starts as ‘To that end, for any given K …’ has a typo as it abruptly ends with the word ‘of’.

We apologize for the typo. It has been fixed.
