# Supplementary material for: SiGMoiD: A super-statistical generative model for binary data
Source: PLoS Comput Biol. 2021 Aug 6;17(8):e1009275. doi: 10.1371/journal.pcbi.1009275 (PMC8372922; doi:10.1371/journal.pcbi.1009275)
Supplement: Attachment — Submitted filename: PLOS_reviews.docx [file pcbi.1009275.s010.docx]

Dear Editors,

We thank both the reviewers for their insightful comments. We have now addressed their issues. Below, we provide a point-by-point response.

Thank you

Xiaochuan Zhao, German Plata, and Purushottam Dixit

**Reviewer 1**

1. We thank the reviewer for this comment. We have now added a sentence stating that in the presence of correlations among data points, one can potentially use cross-validation to decide the number of components (lines 263-265).
2. The collection of N binary variables is imagined as interacting with one *bath* that exchanges K types of energies with the variables.

**Reviewer 2**

Reviewer 2 did not have any suggestions
